# Comprehensive review on how platinum- and taxane-based chemotherapy of ovarian cancer affects biology of normal cells

**DOI:** 10.1007/s00018-018-2954-1

**Published:** 2018-10-31

**Authors:** Justyna Mikuła-Pietrasik, Anna Witucka, Martyna Pakuła, Paweł Uruski, Beata Begier-Krasińska, Arkadiusz Niklas, Andrzej Tykarski, Krzysztof Książek

**Affiliations:** Department of Hypertensiology, Angiology and Internal Medicine, Poznań University of Medical Sciences, Długa 1/2 Str., 61-848 Poznań, Poland

**Keywords:** Chemotherapy, Ovarian cancer, Platin analogs, Side effects, Taxanes

## Abstract

One of the most neglected aspects of chemotherapy are changes, and possible consequences of these changes, that occur in normal somatic cells. In this review, we summarize effects of selected drugs used to treat ovarian cancer (platin derivatives—cisplatin and carboplatin; and taxanes—paclitaxel and docetaxel) on cellular metabolism, acquisition of reactive stroma features, cellular senescence, inflammatory reactions, apoptosis, autophagy, mitophagy, oxidative stress, DNA damage, and angiogenesis in various types of normal cells, including fibroblasts, epithelial cells, endothelial cells, and neurons. The activity of these drugs against the normal cells is presented from a broader perspective of their desirable anti-tumoral effects.

## Introduction

Oncologic patients treated with drugs of various groups exhibit side effects of varying severity, mostly attributed to the deleterious activity on normal cells lying nearby or in some distance from cancerous tissue. These side effects are usually considered from a macro perspective in which the presence of complications itself is more important from the clinical point of view than their triggers and mechanisms [1]. Even in a basic science concentrated around an anti-cancer therapy, effects of drugs or drug candidates are studied predominantly on malignant cells, whereas their simultaneous activity towards normal cells is usually omitted. There are only sparse reports in which possible drug candidates suggested as effective against cancer [2] are investigated in the presence of normal cells, which often provides data about their negative influence [3].

During the research for this review, we noticed that the available literature is extremely poor in reports dealing with effects of common chemotherapeutics at the level of normal cells. This finding encouraged us to collect and discuss the current state-of-the-art regarding alterations in the biology of normal cells subjected experimentally to drugs commonly used in chemotherapy. As an example, we arbitrarily selected pharmacological management of ovarian cancer, choosing to investigate the activity of two platin derivatives, cisplatin and carboplatin, and two taxanes, paclitaxel and docetaxel [4].

## Ovarian cancer and current algorithms of its chemotherapy

Ovarian cancer is the most lethal malignancy of female genital tract. The pharmacology of ovarian cancer depends on a stage of the disease (according to criteria provided by International Federation of Gynaecological Oncology, FIGO) and includes cytoreductive surgery and chemotherapy. When the cancer is diagnosed early, that is in FIGO stage I or II, the optimal cure is surgical tumor debulking without adjuvant chemotherapy after which the 5-year survival rate is around 90%. Patients with advanced disease (FIGO stage III or IV) are recommended to undergo maximal surgical cytoreduction followed by a systemic chemotherapy. This treatment should guarantee the 5-year survival rate for up to 30% of patients. Chemotherapy is also a necessity in patients with a recurrent disease [5].

Initially, chemotherapy of epithelial ovarian cancer was based on the platinum derivative, cisplatin or, later, on its less toxic analog—carboplatin [6]. The discovery of high activity of paclitaxel (Taxol^®^), an active component of tree *Taxus brevifolia*, against ovarian cancer has changed initial recommendations regarding the first-line therapy and made three cycles of the combination of carboplatin with paclitaxel the most optimal setting (“golden standard”) in the treatment of epithelial ovarian cancer [7]. In recent years, some trials have been conducted to reduce some aspects of paclitaxel toxicity (e.g. alopecia and neurotoxicity) and other taxanes combined with carboplatin, in particular, semisynthetic docetaxel, were tested. Results of these trials provided evidence that the combination of carboplatin with docetaxel may be used in patients with high risk of neurotoxicity (at the cost of more myelosuppression), but in remaining patients carboplatin combined with paclitaxel should still be treated as the primary cure [8].

Because the effectiveness of systemic chemotherapy of ovarian cancer is far from satisfactory, several attempts have been made to establish an adequate route of a drug administration. Clinicians’ attention was then focused on the intraperitoneal route which seemed to be more appropriate from the standpoint of anatomical determinants of both primary and metastatic ovarian tumors. At the same time, this route should increase dose intensity delivered to any residual tumor, avoiding additional systemic toxicity. It should be noted, however, that drugs delivered locally display lower penetrability deep into tissue stroma, therefore, it should provide benefits exclusively to patients who have undergone optimal cytoreductive surgery. Indeed, numerous trials revealed clinical benefits when drugs were administrated i.p. instead of i.v., however, often at the cost of increased frequency of hematologic or non-hematologic side effects. Currently, the i.p. chemotherapy is considered an alternative choice concerning the classic i.v. route, albeit the latter is still preferred and a debate about the choice of the drug administration route continues [9].

## Mechanisms of anti-cancer activity of platinum derivatives and taxanes

### Cisplatin and carboplatin

**Fig. 1:**
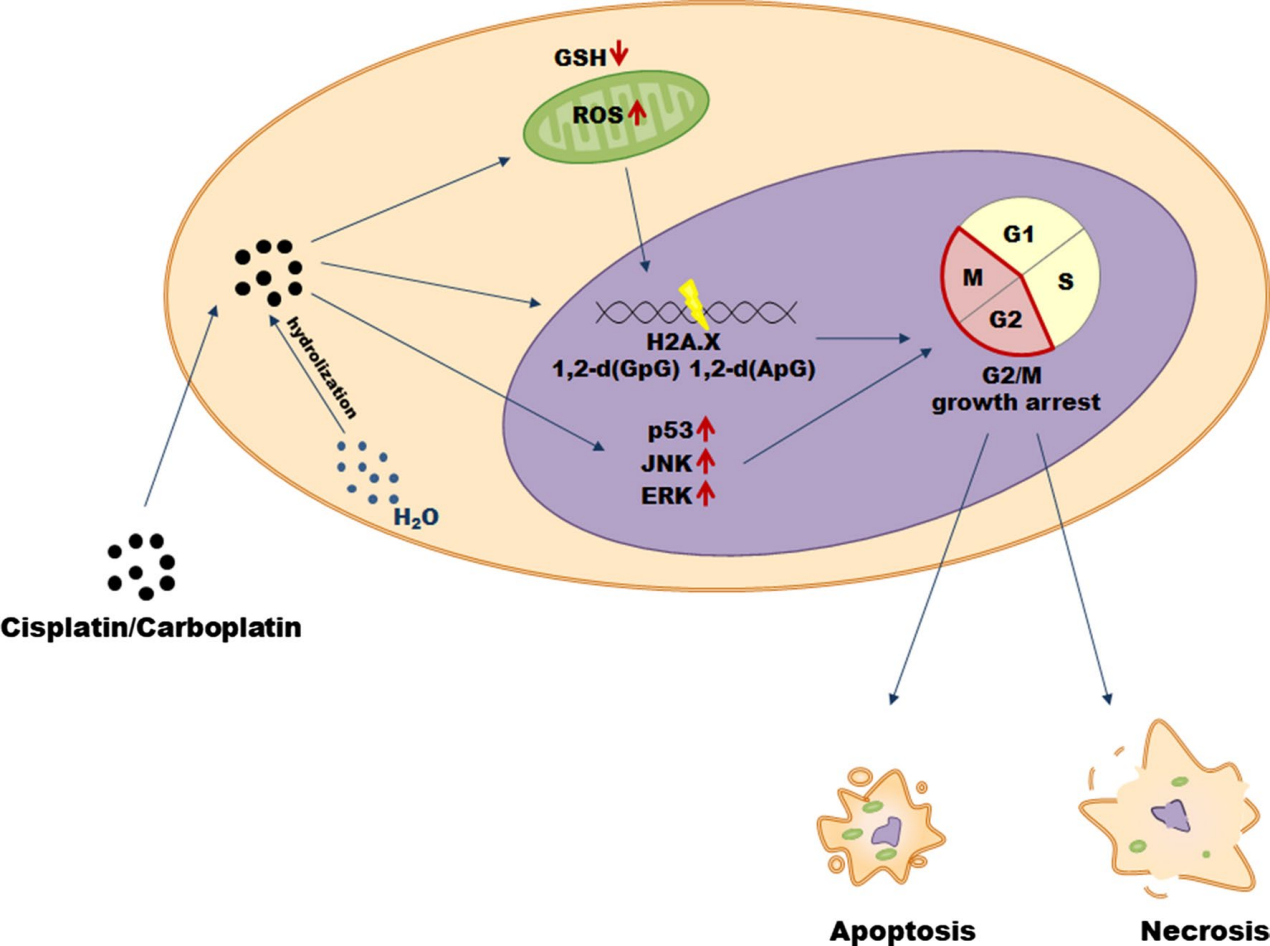
Mechanisms of cisplatin and carboplatin cytotoxicity in cancer cells

Cisplatin (*cis*-diamminedichloroplatinum(II)) is currently one of the most compelling drugs used in cancer chemotherapy (Table 1). Once cisplatin enters a cell, the chloride atoms on the drug are displaced by water molecules, and the hydrolyzed product is capable of reacting with any nucleophile, such as the sulfhydryl groups on proteins and nitrogen donor atoms on nucleic acids. Cisplatin connects to the N7 reactive center on purine bases, which elicits DNA injury that blocks replicative machinery and directs cancer cells towards apoptosis. The spectrum of chemical changes induced by cisplatin within DNA is wide, however, the most prominent is the formation of the 1,2-intrastrand [d(GpG) and d(ApG)] adducts of purines. It has been found that cisplatin may induce DNA damage and cancer cell death also by induction of oxidative stress, particularly the overproduction of mitochondrial reactive oxygen species (ROS) and by decreasing the pool of intracellular antioxidants, e.g. reduced glutathione (GSH). Among signaling molecules and pathways activated in response to cisplatin and involved in the drug-related cytotoxicity, the most important include: p53, extracellular-signal-regulated kinase (ERK), and c-Jun N-terminal kinase (JNK) [10] (Fig. 1).

**Table 1:** Types of malignancies treated with platinum derivatives and taxanes

Group	Drug	Cancer	References
Platinum analogs	Cisplatin	Bladder Cervical Ovarian Gastric Head and neck Testicles Breast Esophageal	[11–17]
	Carboplatin	Lung Ovarian Head and neck Cervical Breast Bladder Testicles Esophageal	[11–16, 18]
Taxanes	Paclitaxel	Breast Ovarian Lung Esophageal Head and neck Bladder Testicles Gastric Cervical	[11, 13–19]
	Docetaxel	Ovarian Breast Lung Prostate Head and neck	[11, 13, 18]

Although approximately 3000 platinum analogs have been synthesized and 13 of them underwent clinical trials, only one of them—carboplatin (1,1-cyclobutyldicarboxylate)—has proved a clinical advantage over cisplatin and thus gained widespread acceptance [20]. Once it penetrates the cell membrane, carboplatin is subjected to hydrolysis becoming positively charged. Similar to cisplatin, it covalently binds to the N7 site of purine bases forming monoadducts or intra and interchain diadducts. The presence of these modifications interferes with cell replication (G2/M growth arrest), leading to cancer cell elimination through apoptosis or necrosis [21].

### Paclitaxel and docetaxel

**Fig. 2:**
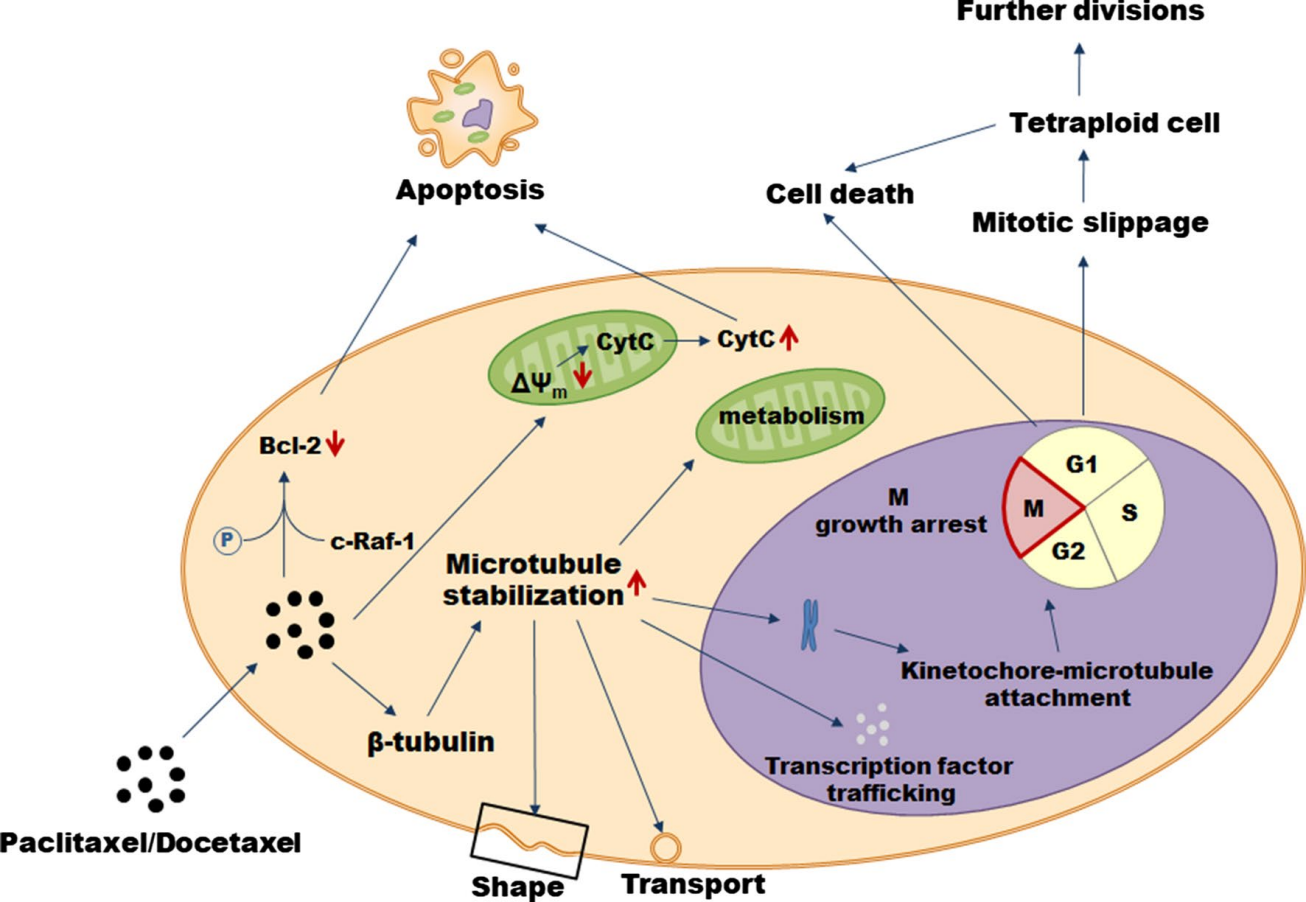
Mechanisms of paclitaxel and docetaxel cytotoxicity in cancer cells

The primary mode of paclitaxel’s action is the hyperstabilization of microtubules—the component of the cytoskeleton composed of repeating subunits of α- tubulin and β-tubulin critical for several cellular behaviors, including: regulation of cell shape, vesicle transport, transcription factor trafficking, mitochondrial metabolism, and the separation of chromosomes during mitosis (Fig. 2). Mechanistically, paclitaxel binds to the N-terminal 31 amino acids of the β-tubulin subunit and decreases the threshold concentration of purified tubulin subunits which are necessary for polymerization into microtubules in vitro and elevates the fraction of tubulin subunits that assemble. Also, paclitaxel interacts directly with microtubules stabilizing them against depolymerization by cold and calcium. As a result, cancer cells treated with the drug are growth arrested in metaphase on bipolar spindles. The effectory role in this process is played by the activation of spindle assembly checkpoint which prevents the progression of cell cycle, in particular, the separation of the chromosomes due to the presence of kinetochores that do not display a solid attachment to microtubules. Cell fates upon the treatment with paclitaxel may be, however, different. One scenario assumes cell death in the course of mitosis while another includes an exit from mitosis without proper chromosome segregation (the so-called mitotic slippage) leading to the formation of a tetraploid G1 cell. As a result of the slippage, the cells may die, remain growth arrested, or undergo further divisions. Mechanisms determining the outcome need still to be explored [22].

One of the best-recognized ways of cancer cell elimination upon the treatment with paclitaxel is apoptosis proceeding in the mitochondria-related mechanism. Cancer cells subjected to the drug are characterized by decreased inner mitochondrial membrane potential (Δ*Ψ*_m_) which leads to the opening of permeability transition pore channel and the release of cytochrome *c* and apoptosis-inducing factor. Eventually, apoptotic death is executed by the activated effectory caspases [23]. Some role in directing cancer cells towards apoptosis is also played by the ability of paclitaxel to phosphorylate and inactivate the anti-apoptotic protein Bcl-2 in a trait related to c-Raf-1 proto-oncogene [24] or, alternatively, via a direct effect on mitochondria [25].

When it comes to docetaxel, its mode of anti-cancer activity is very similar to that of paclitaxel, although it differs structurally from the former at either the 3′ position on the side chain or the 10′ position on the baccatin ring [26]. Specifically, docetaxel also targets microtubules affecting the β-tubulin, however, experiments based on site-directed mutagenesis revealed that the molecular nature of these interactions is slightly different [27]. At the same time, docetaxel has been found to be twice as active in microtubule depolymerization inhibition as paclitaxel [28]. Both paclitaxel and docetaxel are routinely used to treat significant number of tumors (Table 1).

## Biology of normal cells upon treatment with platinum derivatives and taxanes

Both the analogs of platinum and taxanes used in the management of ovarian cancer are known to cause several systemic and local side effects and complications. Drug resistance leading to an insufficient response to a therapy is also common in ovarian cancer patients and often leads to the disease recurrence [29]. In our opinion, it is reasonable to think that issues mentioned above may be associated in a significant degree with deleterious effects of chemotherapeutics on normal, non-cancerous cells and tissues [30].

The most common, irrespective of drug administration route (i.v./i.p) complications of ovarian cancer treatment include: leucopenia, thrombocytopenia, fatigue, nausea, vomiting, diarrhea, hearing loss, nephrotoxicity, neurotoxicity, ototoxicity, gonadotoxicity, and hypersensitivity reactions. Clinical observations also indicate that a group of complications is directly associated with the specificity of intraperitoneal drug delivery, and these include abdominal pain, peritonitis, chills, and catheter migration [31]. The probability and severity of side effects depend on a patient response to a treatment and the therapy regimen used [32].

Currently, the above mentioned adverse results of chemotherapy are well described from a clinical perspective, but at the same time, the knowledge about their biological (cellular and molecular) mechanisms is lagging. Next sections of this review are devoted to processes which occur in various populations of normal somatic cells in response to platinum analogs and taxanes, and which may contribute in our opinion to the side effects of cancer chemotherapy, as well as to a therapeutic failure and/or paradoxical exacerbated progression of the disease. The most important information provided in this section have additionally been summarized in Tables 2 and 3.

**Table 2:** Biological effects of platinum derivatives on normal cells

Activity	Mechanism	Cell type	Drug
Catabolic metabolism	Increased consumption of glucose and generation of lactic acid	Fibroblasts	CIS; CAR
	Induction of autophagy	Tubular epithelial cells	CIS
Drug resistance	Increased expression of anti-apoptotic proteins in cancer cells related to increased production of IL-11 by fibroblasts	Skin fibroblasts	CIS
	Accumulation of drugs in normal cells instead of cancer cells	Skin fibroblasts	CIS
Induction of cellular senescence	Up-regulation of cell cycle inhibitors; deterioration of cell–cell communication; Induction of SA-β-Gal	Skin fibroblasts	CIS; CAR
Induction of pro-inflammatory phenotype	Activation of NF-κB-dependent inflammatory response	Proximal tubule epithelial cells	CIS
	Overproduction of IL-1 and IL-6	Umbilical vein endothelial cells	CIS; CAR
	Overproduction of ICAM-1 and IL-8	Retinal endothelial cells	CAR
	Overproduction of ICAM-1 and ELAM-1	Dermal endothelial cells	CAR
Induction of cell death	Dysfunction of mitochondria; activation of caspases	Renal epithelial cells; endothelial cells	CIS
Induction of oxidative stress	Increased production of ROS; decreased activity of antioxidants; deregulation of mitochondrial metabolism	Renal proximal tubule epithelial cells	CIS
	Increased DNA damage	Hippocampal neurons	CIS
	Increased DNA damage	Fibroblasts; Schwann cells	CAR
Modulation of angiogenesis	Impaired MMP-2-related reactions of vascular endothelium	Endothelial cells	CIS
	Increased production of VEGF	Endothelial cells	CAR

Detailed description of effects summarized in this table is provided in the text

*CIS* cisplatin, *CAR* carboplatin

**Table 3:** Biological effects of taxanes on normal cells

Activity	Mechanism	Cell type	Drug
Catabolic metabolism	Increased consumption of glucose and increased production of lactic acid	Skin fibroblasts	PAC
	Initiation of autophagy	Aortic endothelial cells	PAC
	Inhibition of autophagy	Smooth muscle cells	DOC
Induction of reactive stroma phenotype	Transdifferentiation towards myofibroblasts	Skin fibroblasts	PAC
Inhibition of reactive stroma phenotype	Reduced expression of CAFs’ markers	Renal fibroblasts	PAC
Anti-cancer activity	Induction of cancer cell apoptosis	Lung fibroblasts	PAC
Pro-cancerous activity	Increased adhesion, invasion and proliferation of cancer cells	Breast cancer-associated fibroblasts	DOC
Inhibition of pro-fibrotic effects	Decreased incidence of TGF-β1-dependent development of EMT	Peritoneal mesothelial cells	PAC
	Decreased production of collagen	Gallbladder epithelial cells	PAC
Induction of cellular senescence	Increased expression of SA-β-Gal	Vascular endothelial cells	PAC
	The development of SASP	Skin fibroblasts	PAC
Induction of pro-inflammatory phenotype	Intensification of COX-2-related reactions	Endothelial cells	PAC
	Overproduction of IL-6	Skin fibroblasts	PAC
	Induction of NF-κB	Prostate fibroblasts	DOC
Induction of cell death	Induction of p53-dependent apoptosis	Aortic endothelial cells	PAC
	Activation of caspases	Umbilical vein endothelial cells	DOC
Induction of oxidative stress	Increased formation of ROS; Down-regulation of antioxidative enzymes	Fibroblasts; endothelial cells	PAC; DOC
Modulation of angiogenesis	Inhibition of angiogenic reactions of vascular endothelium	Endothelial cells	PAC; DOC
	Stimulation of VEGF release	Umbilical vein endothelial cells	PAC

Detailed description of effects summarized in this table is provided in the text

*PAC* paclitaxel, *DOC* docetaxel

### General metabolism

The first line of changes which appear to occur in normal cells exposed to chemotherapeutics are abnormalities in their general metabolism, in particular, in cell’s energetics. Studies employing normal stromal fibroblasts subjected to 12 common chemotherapeutic drugs, including cisplatin, carboplatin, and paclitaxel, showed that cells maintained under such a regimen consume much more glucose and generate more lactate, which is then released through overexpressed proton-linked monocarboxylate transporter MCT4, leading to augmented acidification of extracellular environment [33].

Detailed metabolic analysis of stromal fibroblasts treated with paclitaxel was possible thanks to experiments using quantitative proteomic profiling. They revealed that cells exposed to this agent exhibit increased expression of several enzymes engaged in glycolysis (hexokinase, 6-phosphofructokinase type C, glyceraldehyde-3-phosphate dehydrogenase, pyruvate kinase), pentose phosphate route (glucose-6-phosphate dehydrogenase, 6-phosphogluconolactonase), and hexosamine biosynthesis pathway (glutamine–fructose-6-phosphate transaminase 1, glucosamine-6-phosphate deaminase 1). Changes in mitochondrial glucose metabolism included down-regulated expression of enzymes regulating Krebs cycle (citrate synthase, aconitate hydratase, dihydrolipoyl dehydrogenase, fumarate hydratase, malate dehydrogenase) and the course of oxidative phosphorylation (NADH dehydrogenase, succinate dehydrogenase, cytochrome c oxidase, ATP synthase). Ingenuity Pathway Analysis aimed at identifying alterations in canonical metabolic pathways also revealed an activation of peroxisome proliferator-activated receptor α (PPARα) and with retinoid X receptor α (RXRα), which may suggest increased ketogenesis and fatty acid β-oxidation [34].

In addition, an on-line survival analysis tool showed that a catabolic metabolism of fibroblasts treated with paclitaxel correlates with survival of patients suffering from various kinds of cancer. For example, up-regulated expression of ubiquitin-like modifier activating enzyme (UBA1) which is involved in ubiquitin conjugation marking proteins for degradation, strongly correlates with progression-free survival in patients with ovarian cancer subjected to this drug [34]. In our opinion, the changes in the basic cellular metabolism described above may have a special importance because they may causatively contribute, obviously in a different extent, to all more specific abnormalities in normal cell functioning which will be delineated in next sections of this chapter.

### Acquisition of reactive stroma characteristics

Another feature of stromal (skin) fibroblasts subjected to cisplatin, carboplatin, and paclitaxel is an induction of their phenotypic and metabolic transformation into cancer-associated fibroblasts (CAFs) [33]. Further analysis concentrated exclusively on cells treated with paclitaxel showed that they adopt numerous markers of transdifferentiation towards myofibroblasts, including α-smooth muscle actin (αSMA), fibroblast activation protein (FAP), calponin, myosin, talin, and vimentin [34]. As per the significance of this functional shift, a body of evidence suggests that CAFs differ remarkably from normal fibroblasts regarding either molecular characteristics or their capacity to support various elements of cancer progression [35]. Regarding, e.g. the peritoneal carcinomatosis which is typical for advanced stages of ovarian cancer and the recurrent and/or terminal disease (also in patients treated with chemotherapy [36]), CAFs have been found to stimulate both adhesion and invasion of the cancer cells in vitro, as well as tumor growth and dissemination in a mouse xenograft model in vivo [37].

The presence and activity of CAFs may also play a role in drug resistance of cancer cells to cisplatin. The fibroblasts subjected to this drug display an up-regulated production of IL-11, which resulted in diminished cancer cell susceptibility to cisplatin-induced apoptosis. Mechanistically, IL-11 released by cisplatin-treated fibroblasts induced phosphorylation of signal transducer and activator of transcription 3 (STAT3) and elevated the expression of anti-apoptotic Bcl-2 and survivin in cancer cells [38].

This is, however, not the only possible mechanism by which platinum-treated fibroblasts confer cancer chemoresistance. Another route may involve the capacity of fibroblasts to restrict the nuclear accumulation of cisplatin in cancer cells, which is associated with increased level of glutathione and cysteine secreted by these cells to the environment [39]. Experiments using murine embryonic fibroblasts extended these findings by demonstrating that high rate of cisplatin and carboplatin accumulation in fibroblasts (instead of malignant cells) is controlled by the major copper influx transporters CTR1 [40] and CTR2 [41].

Generally, the acquisition of CAFs phenotype applies to fibroblasts exposed to either cisplatin or paclitaxel, however, some effects exerted by these drugs differ, which may depend on cells’ type and drugs’ concentration used. This is the case, for example, for renal interstitial fibroblasts which exposed to paclitaxel showed inhibited tyrosine-phosphorylated STAT3 and reduced expression of CAFs’ indicators, αSMA, and collagen I [42]. Anti-cancerous capabilities of skin fibroblasts treated with paclitaxel were also documented by another group who recorded the capacity of these cells to uptake and subsequent release of paclitaxel in a time-dependent fashion. Significantly, conditioned medium generated by paclitaxel-treated fibroblasts could inhibit the growth of various cancer cell lines in vitro [43]. These observations diversifying some effects caused by cisplatin and paclitaxel agree with results of experiments on lung fibroblasts which generated conditioned medium capable of inducing cancer cell death upon the cell exposure to paclitaxel, but not cisplatin [44]. Above mentioned differences in the activity of fibroblasts exposed to paclitaxel and/or platinum drugs seem to depend on the anatomical origin of these cells. Namely, cells transformed into procancerous CAFs originated from the skin [33, 34], whereas cells exerting anti-cancer activity were obtained from kidneys [42] and lungs [44]. In our opinion, different ranges of paclitaxel’s concentrations used may be also taken into account. Last but not least, these diverse effects may be associated with the issue whether the drug-exposed fibroblasts eventually adopted full molecular and functional CAFs’ characteristics, or not.

As per docetaxel, it potentiates the procancerous potential of fibroblasts. It has been found that breast cancer cells co-cultured with CAFs obtained from patients treated with this drug were characterized by increased adhesiveness, invasion, and proliferation as compared with their counterparts cultured in the absence of the CAFs. This action was plausibly associated with overexpression of several genes involved in tumor progression, such as MCP-1, MMP-1, IL-8, RARRES-1, FGF-1, and CXCR7 in docetaxel-treated fibroblasts [45]. The pattern of cancer-promoting genes differentially expressed in CAFs before and following treatment with docetaxel is, however, much wider [46].

Apart from the contribution of drug-induced CAFs to chemoresistance, cells with myofibroblastic features are also known to promote tissue fibrosis [47]. Taking into account that ovarian cancer chemotherapy usually follows a cytoreductive surgery—which puts the abdominal cavity at risk of the development of intraperitoneal adhesions and fibrosis—the profibrotic effects generated by normal peritoneal fibroblasts due to their exposure to paclitaxel may jeopardize a proper healing of the abdomen and indicate an increased risk of the tissue fibrosis in the future [48]. Intriguingly, the effect of paclitaxel on profibrotic potential seems to be a cell-specific response, possibly limited to fibroblasts. For example, peritoneal mesothelial cells constitute the major fraction of cells within the peritoneal cavity, strongly contributing to the development of intraperitoneal cancer metastases [49]. Experiments performed using peritoneal mesothelial cells showed that their exposure to paclitaxel leads to the inhibition of transforming growth factor-β1 (TGF-β1)/Smad2-dependent epithelial–mesenchymal transition (EMT) of these cells and to decreased expression of mRNA for collagen I. This suggests that modifying molecular characteristics of these cells paclitaxel may prevent tissue fibrosis [50]. Decreased expression of mRNA for collagen I and production of protein was also observed in gallbladder epithelial cells and also this time its activity was based on interrupting TGF-β1 signaling [51].

### Induction of cellular senescence

There is evidence that CAFs may contribute to the formation of a hospitable environment for tumor progression in a route involving cellular senescence. Senescent fibroblasts are known to initiate tumorigenesis [52] and to fuel cancer cell progression both in vitro and in vivo [53]. Some, including our group, believe that senescent fibroblasts are one of the plausible sources of CAFs [54]. When it comes to the activity of platinum-based chemotherapeutics, cisplatin has been found to induce senescence in various cancer cell lines, including hepatoma [55] and nasopharyngeal cancer [56], whereas the similar effect of carboplatin was described in non-small-cell lung cancer [57].

An induction of senescence-associated β-galactosidase (SA-β-Gal), a marker of senescence, has also been reported in stromal fibroblasts subjected to both platinum derivatives. At the same time, the cells displayed an up-regulated level of p53 and p21 cell cycle inhibitors, which are known to act as effectory molecules in senescence-dependent growth arrest. The analogical effect was documented in the same study in case of paclitaxel [33]. Another study revealed that cisplatin-induced senescence of fibroblasts included decreased expression of junctional protein connexin 43 (Cx43), reducing the effectiveness of intercellular communication [58]. The taxane (paclitaxel) has been recognized to induce senescence (elevated SA-β-Gal) also in vascular endothelial cells which coincided with decreased activity of endothelial Nitric Oxide Synthase (eNOS) in these cells [59]. In fact, lowered production of nitric oxide (NO) is considered one of the canonical features of the senescent endothelium [60].

The similarity between CAFs and senescent fibroblasts also encompasses their ability to hypersecrete several pro-cancerous stimuli, which phenomenon is called the senescence-associated secretory phenotype (SASP) [54]. Stromal fibroblasts exposed to paclitaxel exhibit increased production of interleukin 6 (IL-6) [33] and chemokine (C–C motif) ligand 5 (CCL5) [34].

### Inflammation

Chronic inflammation is one of the most fundamental features of cancer, and, of course, it usually develops in a manner independent from the presence of CAFs or other types of senescent cells [61]. The extent of inflammatory reactions becomes often intensified as a result of chemotherapy which may contribute to its failure and even a metastasis development [62]. Cisplatin, for example, is known to induce in ovarian cancer cells NF-κB [63], a transcription factor that controls the production of major proinflammatory agents, such as tumor necrosis factor α (TNFα) and IL-1, IL-6, IL-8 [64].

Cisplatin-related inflammation has causatively been linked with a nephrotoxicity. The migration of cisplatin to kidney cells proceeds via two transporters: a copper transporter Ctr1 [65] and the organic cation transporter OCT2 [66]. Once captured by the kidney proximal tubule cells, cisplatin transforms towards more toxic metabolites (in particular thiols) in a pathway involving the formation of platinum-glutathione conjugates [67]. Importantly, direct comparison of effects exerted by cisplatin and carboplatin revealed that the latter is 20-fold less toxic which has causatively been linked with the completely different route of carboplatin metabolism in the kidney cells [68].

The administration of the drug into mice has been found to trigger the inflammatory reaction via the activation of NF-κB and overexpression of TNFα and cyclooxygenase-2 (COX-2). Moreover, cisplatin induced p53 and caspase-3 in tubular cells leading to their increased apoptosis [69]. Other research showed that kidneys isolated from mice exposed to cisplatin displayed increased expression of transcripts coding for intercellular adhesion molecule 1 (ICAM-1), monocyte chemoattractant protein-1 (MCP-1), heme oxygenase-1, TNF receptor 1 (TNFR1), and TNF receptor 2 (TNFR2). Subsequent in vitro experiments with proximal tubule epithelial cells revealed that cisplatin accelerated the degradation rate of IκB in a time-dependent manner, which may imply that the drug-related proinflammatory response in kidney could be associated with the activation of NF-κB signaling [70].

Quite recently, experiments conducted using mice kidney and tubular cells showed that cisplatin-induced kidney inflammation might be governed by the activation of poly(ADP-ribose) polymerase-1 (PARP-1), an enzyme which in the hyperactivated state decreases the rate of glycolysis and impairs electron transport and ATP formation, which eventually leads to dysfunction and/or death of various normal cell types. Some of these effects have been found to be regulated by up-regulated NF-κB [71]. It has been found that chemical or genetic inhibition of PARP-1 decreases cisplatin-induced overexpression of the adhesion molecules ICAM-1 and VCAM-1 level, leads to a concomitant decrease in the leukocyte and macrophage infiltration [72].

Proinflammatory activity of both platinum analogs has also been demonstrated in case of endothelial cells. Comparative analysis of the magnitude of IL-1 and IL-6 release showed that the production of both these cytokines by umbilical vein endothelial cells is greater in response to cisplatin than to carboplatin [73]. Experiments with retinal endothelial cells showed, in turn, that their exposure to carboplatin results in an increased production of ICAM-1 and IL-8, which was followed by their increased proliferation, migration and apoptosis [74]. The mechanism by which cisplatin up-regulates ICAM-1 involves the activation of NF-κB [75]. Other research on dermal endothelium showed an increased expression of ICAM-1 and endothelial leukocyte adhesion molecule-1 (ELAM-1) which coincided with the infiltration of perivascular space with CD4+ T cells upon their treatment with carboplatin [76]. Proatherosclerotic changes in vascular endothelium exposed to cisplatin also include decreased production of nitric oxide which effect occurs through the inhibition of Akt–eNOS signaling [77].

Augmented inflammation is also a common result of cancer cell treatment with taxanes [78]. As per their effect on normal cells, the proinflammatory activity of paclitaxel has been recognized as an important part of the pathophysiology of drug-induced neuropathic pain. Significant role in this process is played by increased production of ceramide and ceramide-sphingosine 1-phosphate (S1P) in the spinal dorsal horn, which corresponded subsequently with the involvement of S1P receptor subtype 1 (S1PR_1_)-dependent neuroinflammatory reactions, such as: activation of NF-κB and ERK and p38 MAPK kinases, followed by increased generation of TNFα and IL-1β [79].

In endothelial cells, an uptake of paclitaxel leads to an up-regulation of COX-2 activity, which—taking into account the capacity of this enzyme to modulate angiogenesis [80]—significantly reduced endothelial cell proliferation when the drug was combined with COX-2 inhibitor (NS-398). Interestingly, the degree of intracellular accumulation of paclitaxel by endothelial cells appeared to be much greater as compared with other cells types, including fibroblasts, which may explain relatively strong anti-angiogenic effects of this drug and docetaxel in vitro and in vivo [81]. Moreover, endothelial cells treated with paclitaxel combined with TNFα improved the secretion of tissue factor and reduced expression of thrombomodulin and protein C receptor. These changes may indicate that the treatment of cancer patient with paclitaxel may induce TNFα-dependent development of prothrombotic venous milieu, leading to shorter or longer perspective to increased atherosclerosis [82].

When it comes to normal fibroblasts, paclitaxel has been found to up-regulate the production of IL-6 [33]. This effect is possibly associated with the activation of STAT3, whose role as the central element mediating proinflammatory, including IL-6-related, cell response to stress has been well recognized [83]. Other changes in paclitaxel-treated stromal fibroblasts include the overproduction of leukocyte recruiting chemokine CCL5 and the activation of Fcγ receptor-mediated phagocytosis signaling [34].

Docetaxel, in turn, has been found to evoke DNA damage-related induction of NF-κB in prostate fibroblasts, which led to up-regulation of WNT16B, a member of the WNT family of molecules determining cancer cell growth [84], causing finally the attenuation of tumor response to chemotherapeutic and improved tumor growth in mice [85].

### Apoptosis

Apoptosis initiated in normal cells underlies significant part of toxicity produced by platinum analogs. It has been found that upon 12 h after cisplatin administration the caspases 3, 8, and 9 in renal epithelial cells were activated [86]. The role of apoptosis in the development of acute kidney injury due to cisplatin confirmed experiments in vivo with caspase-1^−/−^ mice which appeared to be resistant to the drug-induced kidney failure [87]. As per the mechanism by which cisplatin initializes apoptosis, the main cellular events include an increase in the expression of Bax, the depolarization of mitochondrial membrane, the release of mitochondrial cytochrome c, and the activation of caspase-3. These events are controlled by extracellular signal-regulated kinase ERK1/2, which was evidenced according to their disappearance by renal epithelial cell exposure to inhibitors of the MEK1/2 kinase, which plays the upstream role for ERK1/2 [88]. Other molecular events directing epithelial cells towards cisplatin-induced apoptosis include the up-regulation of Fas and Fas-L [89], activation of DNA damage sensor ataxia telangiectasia and Rad3-related (ATR) and its colocalization with histone H2A.X [90], and the activity of miR-30c, a representative of small non-coding RNA molecules [91]. An important role in this process is also played by p38 mitogen-activated protein kinase (p38 MAPK) whose experimental inhibition produced significantly greater extent of apoptosis. This, in turn, led to the depletion of reduced glutathione (GSH) level and increased drug accumulation in epithelial cells [92].

Interestingly, epithelial cells originating from different regions of a nephron appeared to display diversified sensitivity to cisplatin. Precisely, epithelial cells from the proximal tubule appeared to be more sensitive to cisplatin as compared with an epithelium from distal convoluted tubule, which was attributed to decreased expression in these cells of the anti-apoptotic protein, BCL-X_L_. The study also revealed that sensitivity of renal tubular epithelial cells to cisplatin, irrespective of their origin, is up to 176-times higher as compared with carboplatin [93]. The situations described above resembles the role of anatomical cell localization in the process of CAFs formation in response to a chemotherapy. This may, in turn, support the view that deleterious effects of platinum derivatives and taxanes towards normal cells may be pleiotropic and highly stochastic.

Other normal cells in which cisplatin induces apoptosis are fibroblasts, as exemplified using NIH 3T3 cells [94], and endothelial cells. In the latter, the exposure to cisplatin resulted in a rapid induction of caspase-2 and -3, and cysteine protease, calpain [95]. Further research confirmed an involvement of calpain in the cisplatin-induced injury of endothelial cells [96]. An exposure of breast endothelial cells to cisplatin showed an induction of caspase-3 and -9, and increased poly(ADP-ribose)polymerase (PARP) cleavage [97].

Increased apoptosis has also been documented in endothelial cells subjected to paclitaxel [98] and docetaxel [99].

### Autophagy and mitophagy

Autophagy is a cellular catabolic process in which damaged organelles, protein aggregates, and other macromolecules are degraded in the lysosome [100]. Autophagy has been recognized as a mechanism which may minimize or prevent nephrotoxicity caused by cisplatin [101]. It has been found that the treatment of renal tubular epithelial cells with cisplatin results in a rapid expression of autophagic proteins (LC3-I, LC3-II, Beclin 1 and Atg5) and their formation was related to the activity of tumor suppressor p53. At the same time, cell treatment with p53 inhibitor, pifithrin-α effectively terminated this process [102]. Moreover, it has been observed that the inhibition of autophagy increased magnitude of apoptosis due to activation of caspase-3, -6, and -7 [101].

On the other hand, there are reports that show that the inhibition of autophagy may neutralize nephrotoxicity caused by cisplatin, suggesting that the autophagy may be causatively linked with this process. Such conclusions derive, e.g. from experiments on mice and proximal tubule epithelial cells in which interferon-γ (IFNγ) suppressed cisplatin-induced up-regulation of caspase-3 and restored cells’ viability. At the same time, the cytokine decreased the levels of the markers of autophagy, LC3-II, and p62, and increased the expression of cathepsin D suggesting that IFNγ may support autophagic flux. When the mice were IFNγ-deficient, the apoptosis of tubular cells was much more evident [103].

An initiation of autophagy has also been linked with the activity of paclitaxel. Experiments conducted on human aortic endothelial cells showed that paclitaxel impaired proliferation of endothelial cells in p21-dependent mechanism and inhibited re-endothelialization of scratched endothelium. These effects coincided with an increased incidence of autophagy, as evidenced according to the presence of double-membrane vesicles containing cytoplasmic organelles visualized using transmission electron microscopy and increased expression of LC3B. At the same time, a chemical inhibition of autophagy failed to improve proliferation and re-endothelialization of endothelium implying that dysfunctional motility of these cells upon the treatment with paclitaxel is not connected with exacerbated autophagy [98].

Docetaxel, in turn, has been found to inhibit autophagy (LC3B-II and p62 expression) in pulmonary artery smooth muscle cells. Moreover, the knockdown of Beclin-1 or LC3B with siRNA potentiated docetaxel-induced cell death. The ability of docetaxel to suppress autophagy has been recognized to proceed in a proteasome-dependent manner. Intriguingly, effects exerted by the drug have been considered as beneficial, because in rats with pulmonary arterial hypertension it stimulated the resolution of fibrosis and the regeneration of myocardium [104].

In recent years, a special kind of autophagy, concentrated around mitochondria—mitophagy—has attracted a great deal of attention as another mechanism which may protect renal cells against cisplatin-induced toxicity. In general, mitophagy refers to a process in which dysfunctional mitochondria are degraded to maintain homeostasis of the mitochondrial population and which protects cells against their premature death [105]. There is a consensus that mitophagy plays a role in protecting renal cells against cisplatin-induced toxicity. Experiments using human proximal tubule epithelial cells showed that cisplatin-dependent dysfunction of mitochondria is potentiated by the inhibition of mitophagy with 3-methyladenine. At the same time, the intensification of mitophagy by cell exposure to rapamycin improved mitochondrial function and cell viability upon the exposure to cisplatin. A detailed search for the molecular background of kidney cell protection against cisplatin revealed that the knockdown of dynamin-related protein-1 (Drp1), which is the major regulator of mitochondrial fission, suppressed mitochondrial dysfunction in response to the drug [106]. Another pathway regulating mitophagy in cells exposed to cisplatin includes Pink1/Parkin the knockdown of which induced the deterioration of mitochondrial functioning, leading to elevated cell damage via the inhibition of mitophagy. And conversely, the overexpression of the molecule promoted mitophagy protecting the cells against cisplatin-related mitochondrial dysfunction [107].

### Exacerbation of oxidative stress and DNA damage

Increased oxidative stress is the next, after the elevated apoptosis and inflammation, pathomechanism responsible for kidney cells’ injury in response to cisplatin [108]. It has been found that cisplatin induces the generation of reactive oxygen species (ROS) in renal proximal tubule epithelial cells. Moreover, it repressed the activity of manganese superoxide dismutase (Mn-SOD) and increased expression of p53, p21, Bax and the adaptor protein p66shc in a dose-dependent manner. Significantly, the elevated production of mitochondrial ROS and cell death caused by cisplatin was effectively prevented when the cells were incubated with siRNA against p53 and p66shc. Moreover, the knockdown of p53 restored the activity of Mn-SOD and blocked p66shc, and p53 inhibitor attenuated cisplatin-induced oxidative stress which seems to suggest the prime role of p53 in this process [109]. Other experiments showed that apart from p53, which is an element of DNA damage response, cisplatin induces other elements of this pathways, including ATM, Chk1, Chk2, and Kap1 [110].

Another mechanism by which cisplatin augments oxidative stress is associated with the depletion of reduced glutathione (GSH) content. Accumulation of the drug inside renal epithelial cells leads to its actuation into reactive molecules that interact with thiol-containing molecules including GSH. The inactivation of this compound leads to the accumulation of endogenous ROS, which contribute to augmented cell death through an induction of MAPK kinases, p53 and p21 [108]. Cell cycle inhibitory protein, p21, is also activated in response to cisplatin in skin keratinocytes [111].

The third way by which cisplatin causes oxidative stress-related nephrotoxicity involves dysfunction of mitochondria in which the drug rapidly accumulates. There is evidence that ROS overproduced in response to cisplatin are mostly of mitochondrial origin. Their generation leads to fragmentation of the organelles, disruption of mitochondria membrane potential, down-regulation of mitochondrial stability markers, and depletion of ATP. All these effects were effectively prevented by cell pretreatment with GSH precursor, N-acetylcysteine. This report also confirmed a positive feedback between increased mtROS and p53 activation, because the administration of p53 inhibitor significantly reduced ROS release [112]. Another mitochondria-related pathway by which cisplatin induces ROS is cytochrome P450 system. Experiments on animals revealed that CYP2E1-null mice display a low level of ROS produced upon exposure to cisplatin and their kidneys do not undergo the drug-induced failure [113]. In addition, tests on intestinal epithelial cells, in which cisplatin also up-regulates the production of ROS, revealed that severity of cellular damage to cells exposed to cisplatin is proportional to the density of mitochondria [114].

Recent experiments provided evidence that cisplatin-induced oxidative stress in proximal tubule epithelial cells is associated with the activity of poly(ADP-ribose) polymerase 1 (PARP1). It has been observed that an inhibition of this enzyme with PJ34 hydrochloride restored the activity of antioxidative enzymes (SOD, catalase, and glutathione peroxidase) that was diminished after cell challenge with cisplatin. Significantly, the inhibition of PARP1 also restored decreased expression of sirtuin 3 which appeared to be a key molecule for cisplatin-induced oxidative stress, as its loss in the drug-treated cells inhibited the protective effect of PARP1 inhibition, represented by the concentration of lipid peroxidation products and DNA adduct, 8-hydroxy-2′-deoxyguanosine [115].

It has been observed that cisplatin-exposed rat hippocampal neurons display increased DNA damage, impaired respiratory activity, increased oxidative stress, and activated caspase-9. Importantly, cell exposure to N-acetylcysteine, a precursor of glutathione, prevented cisplatin-induced oxidative stress in vitro and cognitive impairment in laboratory rats in vivo [116]. Neuropathy associated with a treatment with cisplatin also involves direct binding of the drug to mitochondrial DNA (mtDNA) inhibiting its replication, which was evidenced in dorsal root ganglion sensory neurons. Interestingly, the affinity of cisplatin to mtDNA was comparable with that characterizing nuclear DNA. Furthermore, cisplatin reduced mtDNA transcription which was probably associated with the distance the gene is localized from the transcription initiation place, which suggests that randomly formed platinum adducts block transcription [117].

Increased oxidative stress is also a result of carboplatin activity on normal cells which is attributed to the bone marrow suppression and ototoxicity [118]. Neurotoxicity, in turn, is linked with carboplatin-induced DNA damage. It has been found that the drug destroys DNA in rat fibroblasts and Schwann cells in a dose- and time-dependent manner. Direct comparison of cell response to the drug revealed that Schwann cells are more vulnerable collect DNA injury than fibroblasts. Interestingly, toxic effects of carboplatin were delayed in comparison with cisplatin, explaining at least partly diversified neurotoxic potential of both platinum derivatives [119].

Increased oxidative stress also characterizes fibroblasts exposed to paclitaxel. It has been found that paclitaxel increases generation of ROS [33] and down-regulates activity of several antioxidative enzymes in these cells, including superoxide dismutase and glutathione peroxidase [34]. In endothelial cells, paclitaxel induced elevated ROS release and the drug-induced apoptosis was aggravated by their incubation with ROS scavengers suggesting the causative relationship between programmed cell death and oxidative stress [120]. It is also worthy to note that ROS generated by cancer cells treated with paclitaxel accumulate mainly outside the cells which leads to lethal damage also in closely localized cells not exposed directly to the drug [121].

Prooxidative activity has also been described in case of docetaxel, which has been found to increase the generation of ROS in endothelial cells in a mechanism involving increased NADPH oxidase activity and protein kinase C β (PKCβ) phosphorylation [99].

Concluding this section, it should be noted that the characteristics of oxidative stress and DNA injury discussed here should be considered in a broader perspective of molecular events accompanying pro-senescence activity of chemotherapy, in which the activity of ROS, the accumulation of oxidative modifications of macromolecules, and the induction of certain signaling pathways play the pivotal role.

### Impaired angiogenesis

Both platinum drugs and taxanes have been recognized as strong modulators of angiogenesis. It has been found that cisplatin attenuates critical functions of vascular endothelial cells involved in angiogenesis, such as proliferation [122] and migration [123]. The anti-migratory activity of cisplatin has causatively been linked with its ability to reduce the production of matrix metalloproteinase 2 (MMP-2) [123]. Carboplatin, in turn, has been found to stimulate the production of vascular endothelial growth factor (VEGF) by endothelial cells, but cell treatment with VEGF-neutralizing antibodies sensitized the endothelium to carboplatin, which resulted in a massive apoptotic cell death [124].

As per taxanes, paclitaxel has been found to inhibit proliferation, migration, and tube formation by endothelial cells [125]. Significantly, anti-angiogenic effects of paclitaxel have been linked with its cytostatic effects (G_2_-M arrest, increase in Bax/Bcl-2 ratio, and mitochondria permeabilization) that eventually result in apoptosis [126]. Experiments performed on microvascular endothelial cells (HMEC-1) and umbilical vein endothelial cells (HUVECs) showed that, paradoxically, their exposure to paclitaxel increases microtubule overall dynamics, which was accompanied by a slight decrease in anaphase/metaphase ratio. Importantly, this mechanism of paclitaxel activity was valid only at concentrations which inhibited angiogenesis without simultaneous induction of apoptosis [127].

Experimental data regarding the effects of paclitaxel on angiogenesis are not, however, uniform. There is a report in which paclitaxel appeared to induce NF-κB and stabilize hypoxia-inducible factor-1alpha (HIF-1α), which translated to increased production of VEGF [120].

Another mechanism of angiogenesis inhibition, namely events associated with impaired repositioning of the microtubule organizing center, has been described for endothelial cells treated with docetaxel [128]. Moreover, decreased migratory properties of endothelial cells exposed to docetaxel are associated with an ubiquitination and subsequent proteasomal degradation of heat shock protein 90 (Hsp90), which prevents signals from the focal adhesions and inability of endothelial cells to respond to VEGF. In addition, docetaxel inhibited the VEGF-induced phosphorylation of focal adhesion kinase, Akt, and eNOS, whose activity is controlled by Hsp90 [129]. Another mechanism involved in docetaxel-mediated inhibition of endothelial cell motility and adhesion involves suppressed VE-cadherin mediated integrin β1/FAK/ROCK signaling pathway [130]. It should also be mentioned that anti-angiogenic activity of docetaxel also extends to its anti-proliferative effect on vascular smooth muscle cells [131]. The observations presented above, showing both the inhibition and the promotion of angiogenesis indicate that the delivery of oxygen and nutrients to cancerous tissue associated with angiogenesis is a context-dependent phenomenon, and that under certain, unpredictable conditions the progression of tumors may be unwittingly stimulated in an iatrogenic mechanism.

## Strategies to restrict the risk of a chemotherapy-induced toxicity

The increasing knowledge about undesirable effects associated with the use of platinum- and taxane-based chemotherapy towards normal cells and tissues prompted oncologists to develop certain strategies to aimed at minimalizing negative outcomes of the therapy. Probably the most advanced research, based on experiments on laboratory animals and various clinical trials, concerns platinum-induced nephrotoxicity. In this regard, candidate adjuvants include synthetic antioxidants (vitamins C and E, AT1R blocker—losartan, erythropoietin, alpha lipoic acid, N-acetylcysteine, deferoxamine, glutamine), mineral elements (magnesium, selenium, chloride salts, sodium thiosulfate) or a combination of both (CV247—an aqueous mixture of copper gluconate, manganese gluconate, vitamin C, and sodium salicylate) [132]. As per second, common group of side effects related to the use of platinum-based therapy, that is neurotoxicity, preventive strategies include detoxicants (amifostine, sodium thiosulfate), nerve growth factor stimulants (Org 2766, retinoic acid), antioxidants (vitamin E, reduced glutathione), electrolytes, chelators, and ion channel modulators (calcium, magnesium, carbamazepine, oxcarbazepine, nimodipine) [133]. In turn, the prevention of neurotoxicity in patients treated with taxanes has been tested using melatonin, amifostine, antiepileptic agents (gabapentin, pregabalin), corticosteroids (prednisone) and a herbal compound, *Shakuyaku*-*kanzo*-*to* [134]. On the other hand, despite promising findings coming from numerous trials, there are no well-established algorithms of side effect prevention of platinum- and taxane-based chemotherapy in a daily, clinical practice. The problem stems mainly from inconclusive results of clinical tests, often associated with different patients’ responses due to defined (e.g. age, treatment regimen, stage of a disease, cancer histotype) or undefined (genetics?) individual variables. The issue of an effective prevention of the drug-induced cytotoxicity in oncologic patients is, however, highly appreciated and there is an ongoing search for the best patient-oriented solutions.

## Conclusions and perspectives

It is obvious that the number of projects and publications dealing with the effects of platinum-based drugs and taxanes on normal cells is incomparably smaller compared with articles in which anti-cancer activity of these drugs was presented. To some extent, one may understand this disparity because it is determined by the clinical imperative to combat cancer cells as fast and efficiently as possible. On the other hand, last decades provided mounting evidence that deep insight into effects of the chemotherapeutics on normal cells may provide a much wider picture of the biological activity of these drugs. Majority of reports suggests in this regard that normal functioning of non-cancerous cells is then seriously impaired. Significantly, several of these changes in cells’ metabolism and function are clearly procancerous, which sheds new light on pathomechanisms of side effects’ development and therapeutic failures. Such the knowledge also means that further investigations of new classes of drugs, aimed at new molecular targets are an urgent need. At the same time, direct comparative studies of effects exerted by these candidates on cancer and normal cells are highly advisable using both in vitro and in vivo models.
